# Considering the Evidence for Anterior and Laterodorsal Thalamic Nuclei as Higher Order Relays to Cortex

**DOI:** 10.3389/fnmol.2019.00167

**Published:** 2019-07-03

**Authors:** Brook A. L. Perry, Anna S. Mitchell

**Affiliations:** Department of Experimental Psychology, University of Oxford, Oxford, United Kingdom

**Keywords:** anterior thalamus, entorhinal cortex, grid cells, head direction cells, hippocampus, laterodorsal thalamus, prefrontal cortex, retrosplenial cortex

## Abstract

Our memories are essential in our daily lives. The frontal and cingulate cortices, hippocampal system and medial temporal lobes are key brain regions. In addition, severe amnesia also occurs after damage or dysfunction to the anterior thalamic nuclei; this subcortical thalamic hub is interconnected to these key cortical memory structures. Behavioral, anatomical, and physiological evidence across mammalian species has shown that interactions between the anterior thalamic nuclei, cortex and hippocampal formation are vital for spatial memory processing. Furthermore, the adjacent laterodorsal thalamic nucleus (LD), interconnected to the retrosplenial cortex (RSC) and visual system, also contributes to spatial memory in mammals. However, how these thalamic nuclei contribute to memory still remains largely unknown. Fortunately, our understanding of the importance of the thalamus in cognitive processes is being redefined, as widespread evidence challenges the established view of the thalamus as a passive relay of sensory and subcortical information to the cortex. In this review article, we examine whether the anterior thalamic nuclei and the adjacent LD are suitable candidates for “higher-order” thalamic nuclei, as defined by the Sherman and Guillery model. Rather than simply relaying information to cortex, “higher-order” thalamic nuclei have a prominent role in cognition, as they can regulate how areas of the cortex interact with one another. These considerations along with a review of the latest research will be used to suggest future studies that will clarify the contributions that the anterior and LD have in supporting cortical functions during cognitive processes.

## Background

In mammals, interconnected brain regions, including the medial temporal lobes, frontal and cingulate cortices and diencephalon, support the formation of new memories (Aggleton, 2014). An important feature of these extended neural networks is anatomical convergence of cortical and medial temporal lobe connections within the anterior nuclei (ATN) and the laterodorsal nuclei (LD) of the thalamus. Behavioral and physiological evidence also indicate these thalamic structures are important hubs within the memory circuitry. However, how the ATN and LD are influencing this circuitry is not yet well understood.

In humans, damage to the ATN from stroke, alcohol abuse, or neurodegenerative disorders is associated with an impaired ability to form new memories (Harding et al., 2000; Van der Werf et al., 2000, 2003; Carlesimo et al., 2011; Kopelman, 2015; Aggleton et al., 2016; Perry et al., 2018). Animal models with damage to the ATN are also impaired in forming new memories. For example, localized ATN lesions in non-human primates impaired new learning in an episodic-like memory task (Parker and Gaffan, 1997). Similarly, excitotoxic lesions to the ATN in rodents consistently result in severe spatial memory deficits in tasks involving allocentric navigation (Aggleton and Brown, 1999; Mitchell and Dalrymple-Alford, 2005; Aggleton and Nelson, 2015; Dalrymple-Alford et al., 2015; Perry et al., 2018; Wolff and Vann, 2019). Deficits after ATN lesions are not restricted to spatial navigation though. For example, rodents are also impaired at making biconditional discriminations, contextual memory processing, forming fixed paired associations between an object and location, and reproducing accurate temporal order memory for a list of previously presented odors (Sziklas and Petrides, 1999; Gibb et al., 2006; Wolff et al., 2006; Law and Smith, 2012; Dumont et al., 2014). The contribution of the LD to spatial memory has thus far only been explicitly examined in two studies. In one study, LD inactivation resulted in increased reference memory errors in the radial arm maze, and in the other study, excitotoxic LD lesions impaired watermaze acquisition and retention of a fixed platform location (Mizumori et al., 1994; van Groen et al., 2002). Additional causal evidence from rat studies that either combined or extended lesions in the LD with those in the ATN support its role in spatial memory (Warburton et al., 1997; Wilton et al., 2001).

**Figure 1:**
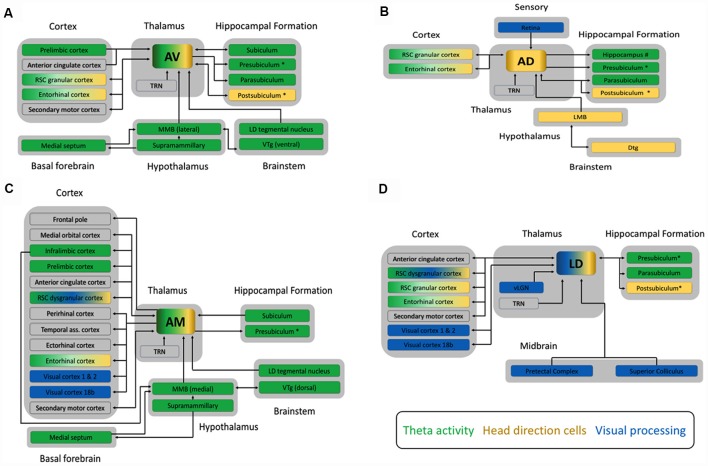
Schematic diagrams outlining the main connections of **(A)** the anteroventral (AV), **(B)** the anterodorsal (AD), **(C)** the anteromedial (AM) subnuclei of the anterior thalamic nucleus, and **(D)** the laterodorsal (LD) thalamic nucleus from studies in rats, cats and monkeys. All four nuclei share dense reciprocal connections to both the RSC and the hippocampal formation. Clear functionally relevant differences are apparent however, between the additional connections of each subnucleus. For example, the AM is broadly connected to many cortical sites including prefrontal, temporal and sensory cortex, whereas the AD has few cortical connections, and does not project to the anterior cingulate like the AM, AV and LD. Another critical point of difference is that all three subnuclei of the ATN receive one primary input containing mnemonically relevant information from the mammillary bodies (MB), whereas the LD receives ascending afferents from regions associated with visual processing, such as the pretectal complex. Arrowheads indicate the direction of information flow, with double headed arrows showing reciprocal connections between structures. The colored boxes indicate the three major functional processes, theta rhythm (green), head direction (gold) or visual processing (blue), associated with these four thalamocortical circuits. Structures associated with two or more of these processes are indicated by a combination of colors. The larger gray boxes group each structure into the broader category of brain region it belongs to, e.g., cortex. Additional connections also exist between cortical structures, the hippocampal formation, midbrain, and brainstem but these are not depicted here. We have also included the presubiculum and postsubiculum as separate structures but we note that the dorsal part of the presubiculum is commonly known as the postsubiculum. Additional abbreviations: Dtg, dorsal tegmental nucleus of Gudden; LD tegmental nucleus, laterodorsal tegmental nucleus; LMB, lateral mammillary bodies; MMB, medial mammillary bodies; RSC, retrosplenial cortex; TRN, thalamic reticular nucleus; vLGN, ventral part of the lateral geniculate nucleus of the thalamus; Visual cortex 18b, Brodmann area 18b; VTg ventral tegmental nucleus of Gudden.

The ATN and LD sit at a convergence point within a complex array of cortical and subcortical connections (Figure 1; Aggleton et al., 2010; Jankowski et al., 2013; Dalrymple-Alford et al., 2015). These include widespread, often reciprocal, links to frontal cortex, cingulate cortex, especially retrosplenial cortex (RSC), and hippocampal formation (Shibata, 1998, 2000; van Groen et al., 2002; Shibata and Naito, 2005). One of the main points of difference between the ATN and LD are the primary subcortical afferents they receive. The ATN receive their primary ascending afferents from the mammillary bodies (MB), which are also strongly implicated in mnemonic processing (Vann, 2010). The inputs to the MB originate in the vestibular system and run *via* the midbrain tegmental nuclei of Gudden (Guillery, 1955, 1956; Taube, 2007). The LD receives its primary ascending afferents from visual structures, including the pretectum, superior colliculus and ventral lateral geniculate nucleus (Thompson and Robertson, 1987).

The ATN can be divided into three subnuclei: anterodorsal (AD), anteroventral (AV), and anteromedial nuclei (AM: Figure 1). Differences in their connectivity have been tied to specific functional distinctions between them (Aggleton et al., 2010). For an excellent description of the anatomical connectivity of the ATN across species, see Bubb et al. (2017). In contrast, anatomical and functional distinctions of the LD are not as well defined, but there is some evidence for a dorsolateral—ventromedial divide (Thompson and Robertson, 1987). The known neuroanatomical connectivity indicates that the LD provides key visual inputs to the extended hippocampal system and entorhinal cortex.

The dorsal aspect of the LD, and the AD are proposed to form part of a lateral head direction circuit along with the postsubiculum, lateral MB, and RSC (Taube, 2007). This circuit is characterized by cells that preferentially fire when the animals’ head is oriented in a specific direction, acting somewhat like a compass. Recent evidence indicates that head direction cells in both the LD and AD coded separately the rat’s heading and movement directions (Enkhjargal et al., 2014). Head direction cells in the LD have been reported to differ from those in the AD, in that they are highly dependent on visual cues, whereas head direction cells in the AD can form highly directional firing after initial exposure to an environment, and can be maintained in the absence of visual cues (Mizumori and Williams, 1993; Goodridge et al., 1998). These differences are likely generated from differences in their respective inputs (Figure 1). The functional implication of these differences is not yet clear, although both types of information are clearly necessary for effective navigation.

In contrast to the LD and AD, the AV and AM are proposed to form part of a theta circuit with the medial MB, prefrontal cortex (PFC), RSC, and hippocampal formation (Vann and Aggleton, 2004; Jankowski et al., 2013). Theta rhythms (3–8 Hz in humans but 4–12 Hz in rodents) within this circuitry are thought to synchronize distally located populations of neurons and provide a framework for the inter-structural communication necessary for complex cognitive functions, such as memory processing (Buzsáki, 2002, 2005; Kirk and Mackay, 2003; Rutishauser et al., 2010; Colgin, 2011). The AV and AM also contain some head direction cells, and the AM some grid-like cells, suggesting they may be important convergence points between multiple streams of information that are filtered and passed on to cortex (Aggleton et al., 2010; Tsanov et al., 2011a,b,c; Jankowski et al., 2015).

Important differences between the LD, AV, AD and AM are also observed in the pattern of cortical connections they receive (Figure 1). This is especially true for the AM, which is linked to many areas of PFC, including medial PFC and anterior cingulate cortex (ACg; Hoover and Vertes, 2007; Xiao et al., 2009; Jankowski et al., 2013). Further differences are found in their respective links with RSC. The AD and AV are predominantly interconnected with granular RSC, which is principally involved in navigational processing, while the AM is predominantly connected with dysgranular RSC, which is principally involved in visual processing (van Groen and Wyss, 1990, 1992, 1995, 2003; van Groen et al., 1999; Shibata, 1998). The LD has reciprocal connections with both granular and dysgranular RSC (Sripanidkulchai and Wyss, 1986; Shibata, 1998, 2000). Further, RSC afferents to the AD, AV and AM originated in layer VI, suggesting that RSC modulates how ATN communicate with other structures, whereas LD receives both layer V (driver) and VI (modulator) inputs. The LD also has reciprocal projections with Brodmann area 18b of the visual cortex, whereas AM only projects to visual cortex (Thompson and Robertson, 1987; van Groen and Wyss, 1992; Shibata and Naito, 2005). Finally, only LD and AV share reciprocal connections with secondary motor cortex, but all four thalamic nuclei project to entorhinal cortex (Shibata and Naito, 2005).

## Summary of the Established Principles

The thalamus sits at an important interface between the cortex and its numerous inputs. Every part of cortex receives a thalamic input, and with few exceptions, i.e., the olfactory input, the thalamus is the sole provider of sensory and subcortical information to cortex (Sherman, 2017). Early studies of trans-thalamic sensory relays suggested almost one to one replication of the primary ascending afferent signal in the thalamus. These findings led to the now entrenched view of the thalamus as a passive relay of information to cortex (Sherman, 2017). In this view, any cognitively relevant transformations of ascending sensory or subcortical information would only occur once they passed through thalamus and reached higher order processing sites in the cortex (Halassa, 2018). In their seminal article, Sherman and Guillery (1996) challenged this simplistic view of thalamic function, suggesting instead that the thalamus contains at least two types of nuclei; “first” order nuclei of sensory or subcortical information as previously proposed, and also “higher” order nuclei that influence cortical activity by supporting the “transfer” of information from one area of cortex to another. Citing a large body of anatomical and physiological evidence on the visual pathway formed by the lateral geniculate nucleus, Sherman and Guillery (1996) demonstrated that even in first order nuclei, the role of the thalamus is highly dynamic with the ability to modulate the information it passes to cortex.

### First Order Nuclei

“First” order thalamic nuclei are those that receive primary ascending afferents or “driver” inputs from peripheral sensory, or subcortical regions (Sherman and Guillery, 1996). One example is the retinal input into the lateral geniculate nucleus of the thalamus, which is “relayed” to visual cortex. First order nuclei also receive distinct fine “modulator” afferents from layer VI of the cortex (Sherman and Guillery, 1996; Sherman, 2016). This modulation is generally linked to the inhibitory GABA pathway passing through the thalamic reticular nucleus (TRN). Modulator inputs form part of a reciprocal circuit, meaning that the layer VI cortical afferents project to the same thalamic region that innervates the layer VI cortical neurons (Sherman, 2016). The “driver” inputs provide the major functional input to the thalamic relay cells and the “modulator” cortico-reticular-thalamic inputs provide a means to “gate” or control the flow of information to cortex (Sherman, 2016).

### Higher Order Nuclei

Unlike “first” order nuclei, “higher” order nuclei receive few or no comparable ascending sensory or subcortical afferents but instead receive two types of afferents from cortex (Sherman and Guillery, 1996). One of these is just like the layer VI modulatory cortico-reticular-thalamic input received by first order nuclei. The other is comprised of coarse afferents from pyramidal cells located in layer V (Sherman, 2016). Therefore, higher order nuclei represent part of a feed-forward cortico-thalamo-cortical pathway that “relays” information from one part of the cortex to another. Interestingly, recent evidence has shown that optogenetic activation of the mediodorsal thalamic nucleus (MD), a higher order nucleus for PFC, does not appear to alter the specificity of cortical representations, but rather enhances the local effective connectivity within the PFC (Schmitt et al., 2017).

Given the prominence of the ATN and LD in memory formation, it is worth considering how they might fit the Sherman and Guillery (1996) model. Such a consideration drives a number of testable hypotheses regarding the functional contribution of ATN and LD to the wider extended hippocampal memory circuit and perhaps could further our understanding of why such profound memory deficits occur when they are damaged. The next section examines the state of our current knowledge with regards to the functional interactions between ATN, LD and their interconnected cortical sites.

## Current State of the Art

The known neuroanatomical differences indicate that rather than considering either the ATN or LD as a whole structure, we should instead consider their subnuclei as separate entities. Previous work has shown that the physiological attributes of the driving inputs to the AD from the lateral MB, and modulatory afferents from cortex implicate it as a first order relay (Petrof and Sherman, 2009). Further, novel molecular evidence has reinforced the functional heterogeneity of ATN subnuclei. Phillips et al. (2018) developed a comprehensive transcriptomic atlas of mouse thalamus. The majority of thalamic nuclei belong to one of three major clusters, which appear to lie on a single continuum relating to the thalamic mediodorsal axis, with any given cortical region getting input from each of these clusters. Interestingly, ATN subnuclei did not cluster together, rather AV along with LD fell into the “primary” cluster. Nuclei within this cluster were enriched in gene encoding neurotransmitters, ion channels, and signaling molecules, all of which contribute to faster channel kinetics and narrower action potentials. By contrast, AM, along with regions like MD, fell into the “secondary” cluster, which were strongly enriched in neuromodulatory genes. There is strong evidence that at least one subnucleus of MD, the parvocellular MD in non-human primates, is a higher order relay for dorsolateral PFC, as it receives inputs from both layer V and VI neurons and appears to modulate intercortical connectivity (Schwartz et al., 1991; Rovó et al., 2012; Mitchell, 2015; Collins et al., 2018). The AM also appears to receive inputs from layer V and VI of the cortex, at least in non-human primates, raising the possibility that it may act as a higher order relay (Xiao et al., 2009), although in rat, it has been categorized as a first order relay (Varela, 2014). Interestingly, the mouse AD did not appear to conform to any of the three clusters defined by Phillips et al. (2018).

Further to these molecular differences, there is growing evidence that ATN is more than a passive relay of hypothalamic and brainstem information to cortex. Recent work has shown how selective manipulations in ATN have a profound impact across many structures in the limbic cortex, likely contributing to the cognitive deficits observed in mammals with ATN damage. For example, temporary inactivation of rat ATN altered grid-like firing patterns of medial entorhinal cortex (MEC) neurons, while ATN lesions reduced the number of grid-cell neurons in the MEC (Winter et al., 2015). This evidence supports the hypothesis that head direction cell inputs from ATN are involved in the formation of MEC grid cell patterns (Winter et al., 2015). Further, viral tracers demonstrated the pathway for head direction information transfer from the AD onto MEC *via* the presubiculum (Huang et al., 2017), with the inhibitory micro-circuity within presubiculum possibly maintaining the head direction signal (Simonnet et al., 2017; Simonnet and Fricker, 2018). In addition, ATN lesions in rats also result in microstructural changes in the hippocampus and RSC (Harland et al., 2014). Along with severe spatial memory impairments, Harland et al. (2014) observed substantial reductions in dendritic spine densities, which are associated with synaptic plasticity in hippocampal CA1 and RSC granular b cortex. Finally, high-frequency stimulation (~130 Hz) of rodent ATN increased neurogenesis in the dentate gyrus and aided performance on memory tasks (Toda et al., 2008; Encinas et al., 2011; Hamani et al., 2011).

Similarly, stimulation of ATN in larger mammals modulated hippocampal field potential in a frequency dependent manner and increased the BOLD response in hippocampus and PFC (Stypulkowski et al., 2014; Gibson et al., 2016); and finally in humans, recordings from multiple depth electrodes in patients with epilepsy showed high-frequency stimulation (~130 Hz) of ATN was capable of decoupling large scale neural networks that included hippocampus, insular cortex, parahippocampal cortex and dorsolateral PFC (Yu et al., 2018).

## Future Directions

**Figure 2:**
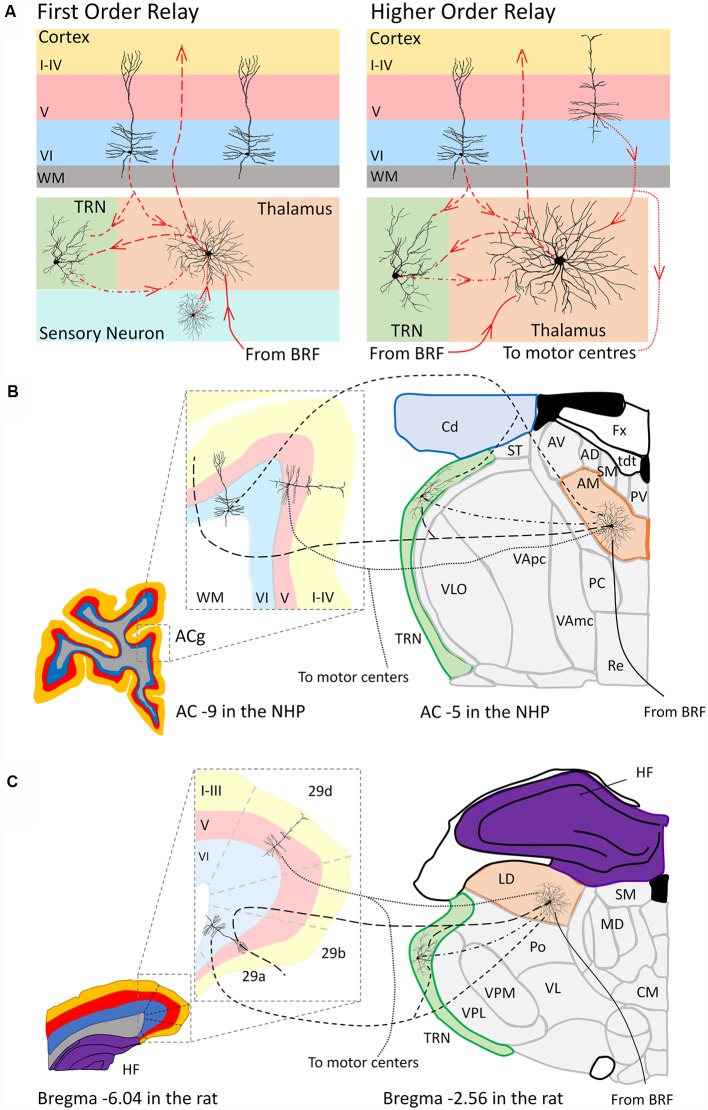
Schematic representation **(A)** of the organization of a first order (left panel) and higher order (right panel) thalamic relay according to the Sherman and Guillery (1996) model. Panel **(B)** depicts a hypothetical scenario based on the work of Xiao and Barbas (2002) and Xiao et al. (2009) of the anteromedial subnucleus (AM, orange) of the anterior thalamic nuclei as a higher order thalamic relay to anterior cingulate cortex (ACg) in the macaque monkey. Panel **(C)** depicts a hypothetical scenario based on the work of Shibata (2000) and Thompson and Robertson (1987) of the laterodorsal thalamic nucleus (LD, orange) as a higher order relay to the dysgranular (29d) retrosplenial cortex in a rat (Shibata, 2000). In a higher order thalamic relay both a “driver” afferent from layer V of the cortex (dotted lines) and a “modulator” afferent from layer VI of cortex (short dashed lines) and the (TRN, green) innervates the thalamic relay neuron. The thalamic relay neuron then in turn projects this cortical information back to layers of cortex (large dashed lines). Projections from the brainstem reticular formation (BRF) and directly from the TRN provide additional modulation to these thalamic relay neurons (Sherman, 2017). Coronal sections for the macaque monkey **(B)** adapted from http://braininfo.rprc.washington.edu/PrimateBrainMaps/atlas/Mapcorindex.html. Images taken at −9 mm and −5 mm from the AC in the macaque brain. Coronal sections for the rat **(C)** adapted from Paxinos and Watson (1998). Images taken −6.04 mm and −2.56 mm from Bregma in the rat brain. Additional abbreviations: 29a-b, Brodmann area 29a-b, granular retrosplenial cortex; 29d, Brodmann area 29d, dysgranular retrosplenial cortex; AC, anterior commissure; AD, anterodorsal subnucleus of the anterior thalamic nuclei; AV, anteroventral subnucleus of the anterior thalamic nuclei; Fx, fornix; Cd, caudate nucleus; CM, centromedial nucleus of the thalamus; HF, hippocampal formation; MD, mediodorsal thalamus; PC, paracentral nucleus; Po, posterior thalamic group; PV, paraventricular nucleus; Re, nucleus reuniens of the thalamus; SM, stria medullaris; ST, stria terminalis; tdt, telodiencephalic fissure; VApc, ventroanterior nucleus (parvicellular); VAmc, ventroanterior nucleus (magnocellular); VI, Layer six of cortex; V, Layer five of cortex; I–IV, Layers one to four of cortex; VL, ventrolateral thalamus; VLO, oral part of the ventrolateral nucleus; VPL, ventroposterolateral thalamus; VPM, ventroposteromedial thalamus, WM, white matter.

Our understanding of cortico-thalamo-cortical interactions and their purpose are still limited, especially with respect to higher order relays. However, based on current findings, there appears to be some evidence that AM (Figure 2) might act as a higher order relay to cortex in primates, while the AD is a first order relay. However, how AV and subnuclei of the LD influence cortex still remain to be fully explored. Influences from outside the limbic circuitry also need to be investigated. For example, inputs from dorsal striatum and medial precentral cortex are likely to be modulating theta within ATN, LD, hippocampal formation and MEC for grid cell formation (Mehlman et al., 2019a,b). Also key to our understanding is whether the relationship of any cortico-thalamo-cortical projections involving ATN and LD are conserved across species. Thus, far cortico-thalamo-cortical interactions have focused heavily on rodent models (Sherman, 2016; Schmitt et al., 2017). Mice and rats provide a great starting point for proof of principle, but they lack the cortical and thalamic development present in higher order species, such as non-human primates and humans (Halassa, 2018). Thus, it is likely that there are differences in fundamental aspects of thalamocortical circuits across species still waiting to be discovered.

What still remains to be understood in neuroscience, and with specific relevance to this review article, is how ATN and LD are managing the various streams of afferent information they receive; clearly the layer VI projections from the RSC are important (Mitchell et al., 2018). Furthermore, it is critical that the nature of the efferent signals they pass on to cortex is characterized. Animal and human experiments that record neural activity from ATN and LD subnuclei and their cortical targets during relevant behavioral tasks will be of great interest. Altering thalamic, striatum, or cortical functioning, using pharmacological agents or optogenetics, and targeting specific cell layers or cell types using transgenic, or viral vector approaches will also be essential to dissecting the specific learning and memory, and navigational functions of these thalamocortical circuits.

Finally, imaging techniques are still constrained by a lack of resolution and continue to struggle to define individual thalamic nuclei (Aggleton et al., 2016). However, using a 7T magnetic imaging scanner and advanced image processing techniques, some of the microstructural components of the MD could be elucidated in humans (Pergola et al., 2018). Consequently, similar strategies may be applied to cognitive and behavioral neuroscience studies investigating ATN and LD, with the caveat that for the ATN at least, it is a much smaller thalamic structure. There has also been increasing work examining ATN-cortical interactions during electrode implant surgeries for refractory epilepsy in humans. We hope that such opportunities will be utilized more in the future, especially in conjunction with detailed cognitive and behavioral tasks and advanced neuroimaging analyses of these patients.

## Concluding Remarks

Evidence from animals and humans support the importance of cortical and subcortical interactions during cognitive processes, including learning and memory, and navigation. Modern neuroscience techniques must now be used to explore how and why these interactions are so critical when we are learning new information, or optimizing our behaviors. In order to advance our knowledge, we must characterize the underlying mechanisms that support these interactions between neural structures important for forming new memories, both in the normal brain, for which animal models remain essential, and in patients with neurodegenerative diseases and neuropsychiatric disorders.

## Author Contributions

BP and AM both contributed to the writing of this manuscript.

## Conflict of Interest Statement

The authors declare that the research was conducted in the absence of any commercial or financial relationships that could be construed as a potential conflict of interest.
